# Prison brain? Executive dysfunction in prisoners

**DOI:** 10.3389/fpsyg.2015.00043

**Published:** 2015-01-30

**Authors:** Jesse Meijers, Joke M. Harte, Frank A. Jonker, Gerben Meynen

**Affiliations:** ^1^Department of Clinical Neuropsychology, VU University AmsterdamAmsterdam, Netherlands; ^2^Department of Criminal Law and Criminology, VU University AmsterdamAmsterdam, Netherlands; ^3^Netherlands Institute for the Study of Crime and Law EnforcementAmsterdam, Netherlands; ^4^Faculty of Philosophy, VU University AmsterdamAmsterdam, Netherlands; ^5^Department of Criminal Law, Tilburg Law School, Tilburg UniversityTilburg, Netherlands

**Keywords:** executive functions, impoverished environment, offenders, prison, recidivism, mini-review

## Abstract

A better understanding of the functioning of the brain, particularly executive functions, of the prison population could aid in reducing crime rates through the reduction of recidivism rates. Indeed, reoffending appears to be related to executive dysfunction and it is known that executive functions are crucial for self-regulation. In the current paper, studies to executive functions in regular adult prisoners compared to non-offender controls were reviewed. Seven studies were found. Specific executive functions were found to be impaired in the general prison population, i.e., attention and set-shifting, as well as in separate subgroups of violent (i.e., set-shifting and working memory) and non-violent offenders (i.e., inhibition, working memory and problem solving). We conclude that the limited number of studies is remarkable, considering the high impact of this population on society and elaborate on the implications of these specific impairments that were found. Further empirical research is suggested, measuring executive functioning within subjects over time for a group of detainees as well as a control group.

## Introduction

More than 11 million people worldwide are in some form detained (Walmsley, 2013), most of them as pre-trial detainees (remand prisoners) or sentenced prisoners. In all continents, the prison population continues to grow (Walmsley, 2013). As the prison population grows, and since crime carries a great (e.g., social and economical) burden (McCollister et al., 2010), reducing recidivism is of great interest to society.

Recidivism rates of prisoners in various countries range between 35 and 67 percent (Langan and Levin, 2002; Spicer et al., 2004; Federal Ministry of the Interior, 2006; Wartna and Nijssen, 2006; Wartna et al., 2012). Several risk factors for recidivism have been identified, such as various demographic risk factors as age and sex (Piquero et al., 2013), unemployment (Verbruggen et al., 2012), and substance abuse (Håkansson and Berglund, 2012). Another risk factor for recidivism is a decline in general self-regulation (Mann et al., 2010) and executive dysfunction (Hancock et al., 2010; Langevin and Curnoe, 2011; Ross and Hoaken, 2011). Executive functions are higher order cognitive functions including planning, working memory, taking initiatives, set-shifting, attention, and impulse control (Jurado and Rosselli, 2007; Diamond, 2013). These functions are crucial for self-regulation (Hofmann et al., 2012). Upon re-entry in society, prisoners face many challenges that place a demand on executive functions. For example, one has to take initiatives, has to be able to plan and think things over, e.g., in order to find housing and employment, both being risk factors for reoffending (Luther et al., 2011). Consequently, executive dysfunction may cause an increase in reoffending, through failure in self-regulation.

In an extensive meta-analysis on the relationship between antisocial behavior and executive functions (Ogilvie et al., 2011), a robust positive association was found between criminality (i.e., people who have committed a crime at some point in their lives) and executive function deficits, with a moderate to large effect size (*d* = 0.6). This relationship between antisocial behavior and executive function deficits has also been clearly shown by other studies, like Morgan and Lilienfeld (2000). However, in these studies heterogenic populations including ex-detainees and patients in forensic hospitals were examined. Even though this may provide useful information for prison policy and (mental) health care, executive (dys)functions in *regular* (e.g., non-hospitalized) adult prisoners have not been reviewed separately. The goal of this systematic review is to provide an overview of studies addressing executive functions in the regular prisoner population.

## Methods

### Search

The aim of our search was to identify studies examining executive functions in regular prison populations, in comparison to non-offender controls. We performed an extensive search in PubMed, Embase, Web of Science and PsycINFO, using a combination of large series of relevant keywords and e.g., MeSH terms (PubMed) or Emtree terms (Embase), such as detained, detention, offenders, executive function and neuropsychological. All prison-related terms were contained within brackets, separated by the “OR” boolean operator, as were all executive function-related terms. The prison-related terms and executive function-related terms were then connected in the final search, as follows: “(prison-related terms) AND (executive function related terms).” The complete search in PubMed, containing all used terms, is provided in Appendix 1 in Supplementary Material. Similar searches were conducted in Embase, Web of Science and PsycINFO. Other relevant articles were found by manually searching bibliographies of already gathered studies.

The combined searches, conducted in February 2013, delivered 1236 results. Two independent reviewers read all the abstracts, which resulted in 190 articles to be read as full text articles. Out of these 190 full text articles, the two independent reviewers found 7 articles meeting all inclusion criteria. An updated search in July 2014 did not produce any additional articles meeting the inclusion criteria.

### Inclusion criteria

Only studies that contained at least one group of adult prisoners and one group of controls (i.e., persons without a history of criminal behavior), which were compared in at least one test of executive functioning, were included. Tests of executive functioning were defined as tests that measured one or more of the following functions: planning, working memory, attention, set-shifting, inhibition/impulse control or problem-solving (Diamond, 2013). Studies were only considered for inclusion if published in the English language. A total of 7 studies fulfilled these criteria and were included in this review. Table 1 provides an overview of the included studies characteristics, like sample size, neuropsychological tasks used and effect sizes.

**Table 1:** **Effect Sizes of Studies with Violent Offenders**.

**Authors (Year)**	**Offender sample**	**Control sample**	**Functions (Tasks)**	**Effect Size (Cohen's D)**
Baker and Ireland, 2007	60, of which:	32 University students	Set-shifting (Benton Word Fluency)	1.09
	42 Violent			0.58
	18 Non-violent			
Greenfield and Valliant, 2007	39, of which:	20 University students	Planning (Porteus Maze)	0.41
	20 Violent			0.06
	19 Non-violent			
Hoaken et al., 2007	40, of which:	20 Respondents to advertising from university and community	Working memory (CSOWM, ASOWM)	N/A
	20 Violent			N/A
	20 Non-violent			
Kavanagh et al., 2010	29 General population	58 From normative sample in Brain Resource International Database	Attention, Set-shifting, Planning, Working Memory (CPT, Letter Fluency, Word Fluency, Maze Reverse Digit Span)	N/A
Munro et al., 2007	15 Violent	15 Facility Staff	Inhibition (Go/No-Go Task)	0.8
Santos Barbosa and Coelho Monteiro, 2008	30 Non-violent recidivists	30 Facility Staff	Inhibition, Planning, Problem Solving (BADS)	0.85–1.66
Schiffer and Vonlaufen, 2011	16 violent	17 respondents to advertising from community	Set-shifting, Planning, Inhibition (WCST, TMT, Word Fluency, Go/No-Go, TOL)	0.52–0.77 (Set-Shifting)
				0.15 (Planning)
				0.03 (Inhibition)

*ASOWM, Abstract Subject-Ordered Working Memory Test; BADS, Behavioral Assessment of the Dysexecutive Syndrome; CPT, Continuous Performance Test; CSOWM, Concrete Subject-Ordered Working Memory Test; TMT, Trail Making Test; TOL, Tower of London; WCST, Wisconsin Card Sorting Test*.

### Effect sizes

Effect sizes (Cohen's D) were manually calculated by the authors where possible, and were classified as small (*d* ≥ 0.2), moderate (*d* ≥ 0.5) or large (*d* ≥ 0.8) (Cohen, 1992). For studies with insufficient data to calculate Cohen's D, effect sizes as reported in the studies were used if available.

## Results

### Set-shifting (3 studies)

Compared to controls, offenders performed significantly worse on set-shifting (Letter Fluency; Baker and Ireland, 2007). Violent offenders in this group performed significantly worse than controls (*d* = 1.09), while the non-violent offenders did not differ significantly, although a moderate effect size was found (*d* = 0.58). Recent findings suggest that certain tasks, e.g., letter fluency, differentiate better between offenders and controls than others (Schiffer and Vonlaufen, 2011). More specifically, moderate effect sizes, implying worse performance of the offenders, were found (*d* = 0.52–0.76) on various other measures of set-shifting (Category Fluency, Wisconsin Card Sorting Test, Trail Making Test), although the differences were not statistically significant. In a study comparing offenders detained for impulsive drug-related crimes with a control group (Kavanagh et al., 2010), no significant effects were found for both Letter Fluency and Word Fluency. In sum, based on the moderate to large effect sizes, it appears that set-shifting may be impaired in both violent and non-violent offenders.

### Planning (4 studies)

As with set-shifting, different planning tasks yield inconsistent results. Non-violent offenders performed significantly worse than controls on several planning subtasks of the Behavioral Assessment of the Dysexecutive Syndrome (BADS; Santos Barbosa and Coelho Monteiro, 2008), with large effect sizes (*d* = 1.00–1.66). However, using the Porteus Maze Test, another planning task, non-violent offenders did not show significantly worse performance than controls (Greenfield and Valliant, 2007), with a negligible effect size (*d* < 0.1). In the same study, a moderate effect size (*d* = 0.41), implying worse performance, was found for violent offenders, although this difference with controls was also not statistically significant. Similarly, in another study using a similar type of Maze test (Kavanagh et al., 2010), no significant differences were found between offenders detained for impulsive drug related crime and controls. A lack of significance was also observed after administering the Tower of London to violent offenders (Schiffer and Vonlaufen, 2011); the effect size (*d* < 0.2) between violent offenders and controls was small.

These findings suggest that only a planning task with an ecological value (BADS; Norris and Tate, 2000), differentiates between offenders and controls. In other words, the BADS may be a better predictor of actual functioning than the other, classic neuropsychological tasks. Inconsistent findings may furthermore be caused by differences between the study populations regarding the rates of recidivism, as more (pronounced) dysfunction could be expected in recidivists (Hancock et al., 2010; Langevin and Curnoe, 2011).

### Working memory (2 studies)

Three tests, primarily appealing to working memory, were administered to violent and non-violent offenders and controls (Hoaken et al., 2007). Of note is that in this study a single unitary executive function variable was created of these three measures. Both violent and non-violent offenders showed significantly worse working memory performance, compared to the control group. In a study comparing offenders detained for impulsive drug-related crime with controls, no difference was found in the Backward Digit Span task (Kavanagh et al., 2010). It should be noted, however, that the Backward Digit Span task is a much less comprehensive task than those used in the first study.

### Inhibition (3 studies)

Three studies assessed inhibition in offenders. In two of these studies inhibition was assessed using a Go/No-Go task in violent offenders (Munro et al., 2007; Schiffer and Vonlaufen, 2011). The first study (Schiffer and Vonlaufen, 2011) found no difference in performance between the offenders and controls (*d* < 0.1). Of note is that the authors reported that their sample consisted of prisoners who mainly had a history of instrumental, rather than impulsive violence, already indicating a lower risk of reduced impulse control. In contrast, the latter study (Munro et al., 2007) did find significantly worse performance of the violent offenders (*d* = 0.8). As both studies had similar sample sizes, similar intelligence values in both the offender and control groups, and controlled for the influence of age and years of education, the inconsistent findings are most likely due to the non-impulsive nature of the participants of the first study (Schiffer and Vonlaufen, 2011).

In the study of Santos Barbosa and Coelho Monteiro (2008), inhibition (using a subtask of the BADS) was significantly worse in non-violent offenders, compared to 30 controls, with a large effect size (*d* = 0.85; Santos Barbosa and Coelho Monteiro, 2008). As mentioned before, the sample in this study mainly consisted of recidivists, and it should be noted that recidivism appears to be related to worse executive functioning (Hancock et al., 2010; Langevin and Curnoe, 2011).

In sum, the findings suggest impaired inhibition in both violent and non-violent offenders, with the exception of those prisoners characterized by a history of premeditated, non-impulsive violent crimes.

### Attention (1 study)

Only one study assessed attention in offenders (Kavanagh et al., 2010). The offenders, a random general sample of offenders, performed significantly worse than controls on the attention task, as the authors report a semipartial correlation of 0.38.

### Problem solving (1 study)

Using the Action Program subtask of the BADS, no significant difference in problem solving outcome was found in 30 non-violent offenders, compared to 30 controls (Santos Barbosa and Coelho Monteiro, 2008). However, the offenders did need significantly more time to achieve the same outcome (i.e., solve the problem), with a moderate to large effect size (*d* = 0.77).

## Discussion

In view of the millions of prisoners worldwide, and the high impact of this population on society, it is striking that only 7 studies were found that examined the executive functions of the general prison population. Furthermore, most studies assessed only some specific executive functions instead of a wide range, and sample sizes were small. Consequently, the results of these studies should be considered with caution. Nevertheless, the findings suggest the existence of various executive function deficits in regular prisoners.

Distinct executive function deficits were found in attention, set-shifting, working memory, problem-solving and inhibition, of which the possible consequences of the consistently found executive dysfunctions will be addressed.

One executive function that was found impaired is inhibition. Inhibition comprises deliberately suppressing ones dominant responses or impulses (Miyake et al., 2000), e.g., in order to think before acting aggressively (Brower and Price, 2001). Prisoners may have difficulties suppressing harmful impulses, such as aggressive impulses.

Set-shifting comprises the ability to change perspectives (Diamond, 2013), for example to think of new solutions for persisting problems, or switch from dysfunctional behavior to more functional behavior. The impaired set-shifting found in prisoners therefore suggests that they may experience increased difficulties to desist from old dysfunctional behavior and to think of other, more effective solutions to their problems.

Working memory provides the ability to actively hold information in mind, and work with that information, for example to keep a certain goal in mind and integrate new relevant information, while discerning irrelevant information (Diamond, 2013). The impaired working memory found in both groups may contribute to a decline in the ability to work toward aforementioned, relatively complex goals, such as finding housing and employment.

Problem solving was found impaired in non-violent offenders. This finding indicates difficulties with solving problems that will arise upon re-entry in society. No studies assessed problem-solving in violent offenders.

As postulated by Miyake et al. (2000), “executive functions are separable but related functions that share some underlying commonality.” In this review, the specific (separable) executive function deficits that were found, are all related to goal-directed behavior and may thus all result in similar problems. We therefore hypothesize that executive dysfunction in regular prisoners has important implications for future reoffending. Further research on this important topic is clearly required.

Out of various treatment strategies, e.g., sanctions and supervision, rehabilitation treatment and cognitive behavior interventions, cognitive behavior interventions focusing on improving specific cognitive skills (e.g., inhibition) were found to be the most effective in decreasing recidivism (Lipsey and Cullen, 2007). However, the results are still unsatisfactory (Ross and Hoaken, 2010) and broad implementation of these interventions is lacking (Lipsey and Cullen, 2007). One might wonder what the effect of comprehensive enrichment of the prison environment itself would lead to, as opposed to occasional treatment. Executive functions have been shown to be positively related to environmental enrichment, e.g., increased physical activity improves executive functions in all age groups (Kramer et al., 1999; Colcombe and Kramer, 2003; Hillman et al., 2008), in particular in those who are sedentary (Scherder et al., 2013). Besides sensorimotor stimuli (physical activity), such comprehensive enrichment of the environment could also comprise enhanced cognitive (e.g., education, occupation) and social challenges (Petrosini et al., 2009). In addition, executive functions of prisoners could be individually assessed, allowing prisons to offer more specific training for prisoners with specific executive function impairments (Ross and Hoaken, 2010).

Prison, however, is currently a clear example of an *impoverished* and sedentary environment. Prison life is characterized by a lack of demand on self-regulating functions, e.g., prisoners are barely confronted with choices to make and have little control over their daily activities (Woodall et al., 2013). Moreover, prisoners may experience resistance from an inflexible prison system, for example when they take the initiative to seek specific health care treatment (Stoller, 2003). In addition, prisoners spend most of their time with passive leisure activities, such as watching television (Elger, 2009). Studies in various countries show that prison is characterized by physical inactivity (Young et al., 2005; Ireland and Culpin, 2006; Cashin et al., 2008; Plugge et al., 2009). For example, prisoners sit or lie on their beds for a striking 9.36 h per day on average, besides the hours spend sleeping (Ireland and Culpin, 2006). As we know from animal studies, an impoverished environment has a negative effect on the prefrontal cortex (Winterfeld et al., 1998; Melendez et al., 2004; Bagorda et al., 2006; Witte et al., 2007), a brain region crucial for executive functions (Jurado and Rosselli, 2007). In the elderly, impoverishment in the form of physical inactivity is related to decreased self-regulation, i.e., increased agitation (Scherder et al., 2010). Thus, the current impoverished prison environment may diminish executive functions and, indirectly, lead to increased recidivism rates. At the same time, an enriched environment may be beneficial to these functions and, in the end, enable successful re-entry in society.

This review shows an emerging field of research that has yet to develop, since large studies with matched healthy control groups are lacking. This development may be challenged by the unique obstacles researchers encounter when conducting research in the prison environment (Vanderhoff et al., 2011), for example logistical difficulties (e.g., lack of testing space, insufficient testing time) or safety issues (e.g., lack of available security staff, obligatory handcuffs during testing). However, as crime carries a considerable (financial) burden to society (McCollister et al., 2010), it is a field with great societal relevance.

In conclusion, the reviewed studies suggest various executive dysfunctions in regular prisoners. This may be due to the higher chance of impairment in antisocial individuals (Ogilvie et al., 2011), deterioration of executive functions caused by the prison environment, or a combination of both. Either way, we hypothesize that the impoverished prison environment, depriving its population of many normal stimuli, may lead to (further) deterioration of executive functions. Within the view that executive functions are crucial for successful re-entry in society, it is imperative that the possible influence of the prison environment is further researched. For future studies to the influence of the prison environment on executive functions, we advise recruiting new detainees for a baseline measurement, and reassessing these new detainees within a certain timeframe, e.g., 3 or 6 months. Combined with a non-offender control group at baseline, it is possible to distinguish dysfunctions that were already present upon imprisonment (which may also have contributed to the imprisonment itself) and dysfunctions caused or worsened by the prison environment.

### Conflict of interest statement

The authors declare that the research was conducted in the absence of any commercial or financial relationships that could be construed as a potential conflict of interest.
