# Supplementary material for: Prison brain? Executive dysfunction in prisoners
Source: Front Psychol. 2015 Jan 30;6:43. doi: 10.3389/fpsyg.2015.00043 (PMC4311616; doi:10.3389/fpsyg.2015.00043)
Supplement: Supplementary file 1 [file DataSheet1.DOCX]

# Appendix 1 – PubMed Search Terms

(antisocial[TiAb] OR "antisocial personality disorder"[TiAb] OR "antisocial personality"[TiAb] OR confinement[TiAb] OR correctional[TiAb] OR criminal[TiAb] OR criminals[TiAb] OR detained[TiAb] OR detainees[TiAb] OR detention[TiAb] OR imprisoned[TiAb] OR incarcerated[TiAb] OR incarceration[TiAb] OR jail[TiAb] OR offenders[TiAb] OR "penal institution"[TiAb] OR penitentiary[TiAb] OR prison[TiAb] OR prisoners[TiAb] OR remand[TiAb] OR Prisons[Mesh:NoExp] OR Prisoners[Mesh] OR Criminals[Mesh] OR Criminal Psychology[Mesh:NoExp] OR Antisocial Personality Disorder[Mesh])

AND

("executive functions"[TiAb] OR "executive function"[TiAb] OR neuropsychologic[TiAb] OR neuropsychological[TiAb] OR neurocognitive[TiAb] OR "neuropsychological test"[TiAb] OR neuropsychology[TiAb] OR "impulse control"[TiAb] OR "tower of london"[TiAb] OR "wisconsin card sorting"[TiAb] OR "stroop task"[TiAb] OR Executive Function[Mesh] OR Impulse Control Disorders[Mesh] OR Neuropsychological Tests[Mesh])
